# A Cartography of Siglecs and Sialyltransferases in Gynecologic Malignancies: Is There a Road Towards a Sweet Future?

**DOI:** 10.3389/fonc.2018.00068

**Published:** 2018-03-13

**Authors:** Quentin Haas, Cedric Simillion, Stephan von Gunten

**Affiliations:** ^1^Institute of Pharmacology, University of Bern, Bern, Switzerland; ^2^Department for BioMedical Research (DBMR), University of Bern, Bern, Switzerland

**Keywords:** gynecologic malignancies, sialyltransferases, sialic acid binding immunoglobulin-like lectins, The Cancer Genome Atlas, cancer immunotherapy

## Abstract

Altered surface glycosylation is a key feature of cancers, including gynecologic malignancies. Hypersialylation, the overexpression of sialic acid, is known to promote tumor progression and to dampen antitumor responses by mechanisms that also involve sialic acid binding immunoglobulin-like lectins (Siglecs), inhibitory immune receptors. Here, we discuss the expression patterns of Siglecs and sialyltransferases (STs) in gynecologic cancers, including breast, ovarian, and uterine malignancies, based on evidence from The Cancer Genome Atlas. The balance between sialosides generated by specific STs within the tumor microenvironment and Siglecs on leukocytes may play a decisive role for antitumor immunity. An interdisciplinary effort is required to decipher the characteristics and biological impact of the altered tumor sialome in gynecologic cancers and to exploit this knowledge to the clinical benefit of patients.

Breast cancer and other gynecologic malignancies, involving the uterus and ovaries, are widely diagnosed tumor entities and, besides lung cancer, constitute the major cause of cancer-related death in women worldwide (1, 2). Although the development and implementation of novel strategies in the treatment of these diseases have positively influenced patient prognosis, novel and more personalized treatment approaches for the heterogeneous forms of gynecologic malignancies are urgently required (3–5). In the past decade, based on scientific evidence, immunotherapies rose to prominence as strong contenders in the fight against cancer in women (3, 6, 7). Different immunotherapeutic strategies are currently under evaluation, whereby off-target immunostimulatory effects of conventional chemotherapeutics may be synergistically embraced in combinatorial immune (chemo) therapeutic regimens (8). However, tumor-intrinsic and -extrinsic resistance factors account for heterogeneous treatment responses (9). The critical importance to decipher these immunosuppressive mechanisms is also illustrated by the unprecedented success of immune checkpoint blockade using antibodies to target immune regulatory checkpoints, such as the inhibitory receptors, CTLA-4 and PD-1 (10, 11). The elucidation of mechanisms that influence the host immune system, in particularly reference to specific gynecological cancers, may lead to novel diagnostic biomarkers and therapeutic strategies for these particular tumors.

Glycosylation changes are common in malignancies (12, 13), and several carbohydrate tumor markers are diagnostically exploited as biomarkers, such as the CA 125 antigen that is elevated in serum of patients with ovarian cancer (14). Distinct patterns of tumor surface glycosylation, in particular, hypersialylation, and the overexpression of sialic acids (15, 16), have been linked to immune escape and tumor progression (12). It has been suggested that sialic acid containing glycans (sialoglycans) may act as “self-associated molecular patterns (SAMPs)” (17) and that hypersialylation of tumors promotes escape from host immune responses by demonstrating “super-self” (18). The recognition of sialoglycan SAMPs by inhibitory receptors on the surface of immune cells, such as sialic acid binding immunoglobulin-like lectins (Siglecs) (19–21), may then lead to the downregulation of immune responses. Members of the Siglec family are heterogenously expressed in immune cells in a cell type- and differentiation-dependent manner, whereby different members recognize structurally distinct sialoglycans (19–21). As a further mechanism, hypersialylation might “mask” glycan ligands of other immunomodulatory receptors, if sialic acids are covalently linked by sialyltransferases (STs) to respective binding sites (12). For instance, the NKG2D-activating receptor on natural killer (NK) cells was shown to be involved in interactions with desialylated ligands on tumor cells (22). Interestingly, it appears that glycan epitopes with terminal sialic acids are less immunogenic and may escape humoral IgG responses (23). Cancer hypersialylation often involves the increased generation of sialoglycan ligands of selectins, such as sialyl-Lewis X and its structural isomer sialyl-Lewis A, which promotes metastatic spread by heterotypic interactions between cancer cells, leukocytes, and endothelial cells (12, 24, 25). In this context, high expression of sialyl-Lewis X in estrogen receptor (ER)-positive breast cancers was reported to correlate with metastasis to the bone, where sialyl-Lewis X receptor E-selectin is constitutively expressed (26). Interestingly, contrarily to this report, sialyl-Lewis X expression was shown to negatively correlate with progression in a breast cancer animal model (27). Many other mechanisms have been reported that contribute to immune escape and progression of hypersialylated tumors (12, 15, 28).

Hypersialylation in cancer has been linked to the enhanced expression and activity of STs (29, 30), which catalyze the covalent attachment of sialic acids *via* different glycosidic linkages (α2–3, α2–6, or α2–8) to subterminal carbohydrate moieties. Notably, high α2,3-sialyltransferase type I (ST3Gal I) expression is associated with advanced stage epithelial ovarian cancer and has been linked to ovarian cancer cell migration and peritoneal dissemination *via* an epidermal growth factor receptor-dependent mechanism (31). Figure 1 provides an overview on the expression of the 20 known human STs in gynecological cancers, including breast carcinoma (BRCA), ovarian serous cystadenocarcinoma (OV), uterine corpus endometrial carcinoma, and uterine carcinosarcoma (UCS), as well as non-gynecologic colonic adenocarcinoma (COAD) based on data from The Cancer Genome Atlas (TCGA) project. Broad, yet differential expression profiles of STs are found at tissue RNA levels across these tumor entities. In BRCA, ST3Gal I (mentioned above) exhibits highest RNA levels on average, followed by ST6Gal I, ST6GalNAc VI, ST6GalNAc II, and ST3Gal IV. Expression levels of these STs are similar in other tumors with the exception of ST6GalNAc II, which were lower in UCS and COAD. In BRCA, particularly low RNA levels are found for ST6GalNAc I, as opposed ST8Sia VI levels are higher. Compared to COAD, most gynecological tumors express at higher levels of ST6GalNAc II, ST8Sia II, and ST8Sia V, but lower levels of ST6GalNAc I.

**Figure 1:**
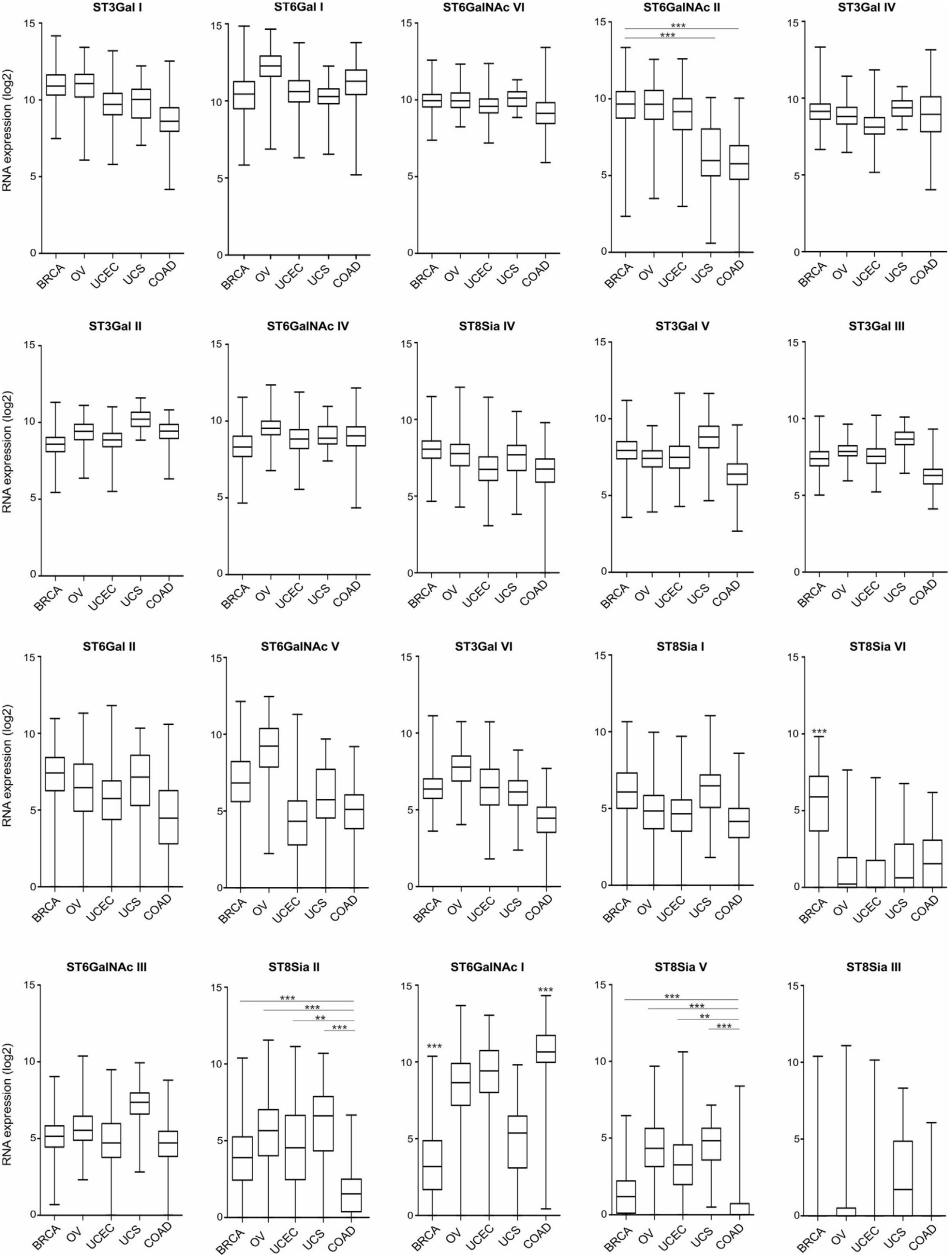
Tissue RNA expression of sialyltransferases (STs) in gynecologic cancers. RNA tissue expression of known human STs in breast carcinoma (BRCA; *n* = 1,094), ovarian serous cystadenocarcinoma (*n* = 305), uterine corpus endometrial carcinoma (*n* = 545), uterine carcinosarcoma (*n* = 57), and colon adenocarcinoma (COAD; *n* = 455), ranked upon expression in BRCA. Data are expressed as box-and-whisker diagrams (median, lower, and upper quartiles; horizontal lines define minimum and maximum). The results shown here are in whole or part based upon data generated by the The Cancer Genome Atlas Research Network: http://cancergenome.nih.gov/. Figures were created in R version 3.4.2. **p < 0.01, ***p < 0.001, one-way ANOVA followed by Bonferroni’s post-test.

Although the exact expression of ST patterns and their consequences remain to be explored, the TCGA data suggest that common patterns of ST expression occur in gynecological tumors that may lead to universal tumor-associated carbohydrate antigens, e.g., sialyl-Tn antigen (14). On the other hand, ST expression differences may contribute to divergent tumor behavior, including immune escape or dissimilar responses to immunotherapeutic interventions. Depending on ST specificity and activity, responses of specific leukocyte subsets may be downregulated upon interaction with a hypersialylated tumor microenvironment, that harbors the cognate sialoside ligands, including specifically sialylated glycoproteins (e.g., mucins) or glycolipids (gangliosides) (12, 19, 24). A number of studies have shown that sialylated tumor cells exploit Siglec receptors to escape immune responses using the sialic acid–Siglec axis, including Siglec-7 or -9 on NK cells (32, 33), or Siglec-9 on myeloid cells (34, 35). The moderate (e.g., Siglec-7 or -9) or even high (e.g., Siglec-2 or -10) RNA expression in gynecological cancers as revealed by TCGA data (Figure 2), indicates the presence of leukocyte subsets that are potentially inhibited by tumor hypersialylation, as a consequence of increased ST expression and activity. Sialic acid–Siglec interactions may thus have important implications in terms of immune escape and for immunotherapeutic strategies in gynecological tumors.

**Figure 2:**
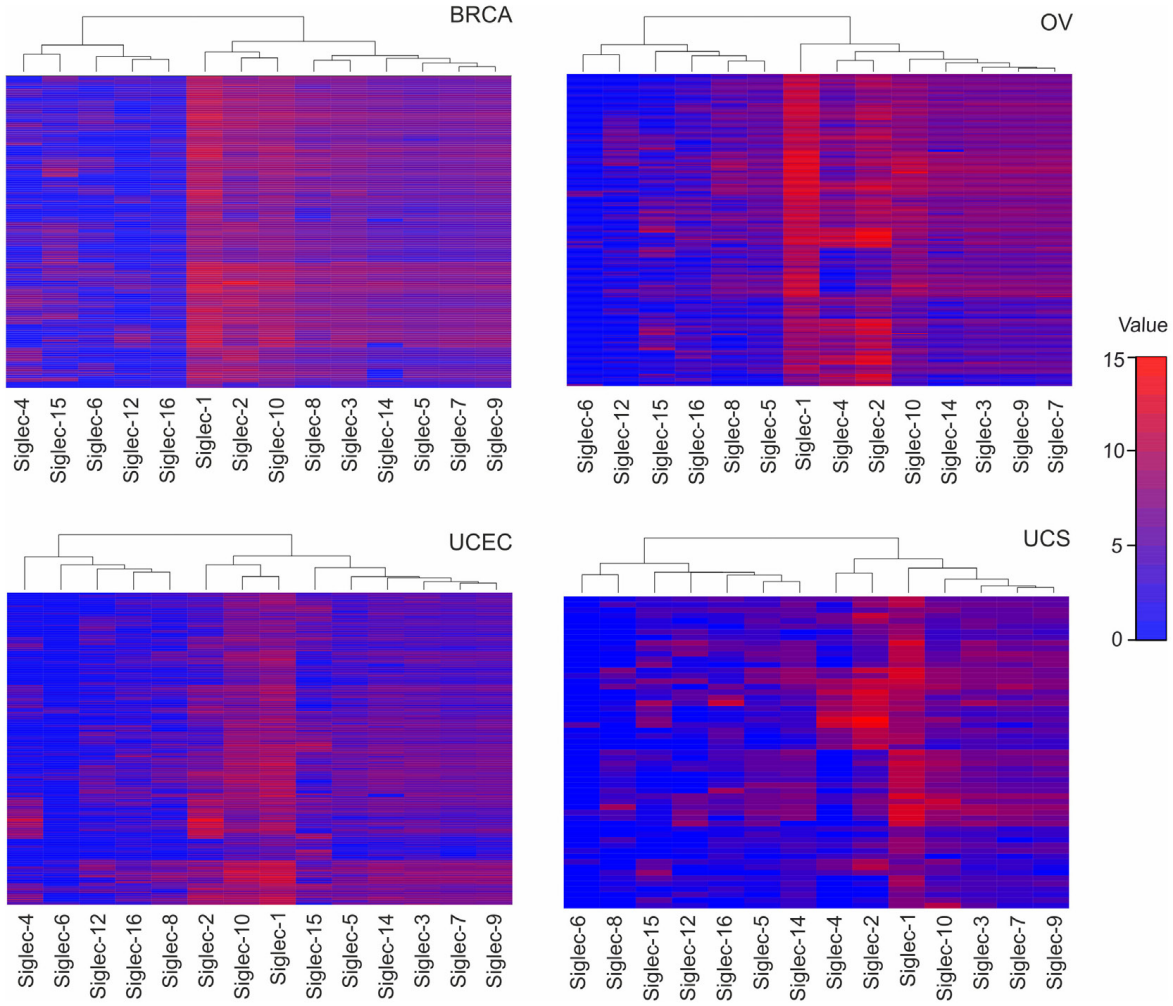
Tissue RNA expression of sialic acid binding immunoglobulin-like lectins (Siglecs) in gynecologic cancers. RNA tissue expression of Siglecs in breast carcinoma, ovarian serous cystadenocarcinoma, uterine corpus endometrial carcinoma, uterine carcinosarcoma, and colonic adenocarcinoma, computed by a dendrogram clustering algorithm in R version 3.4.2. The results shown here are in whole or part based upon data generated by the The Cancer Genome Atlas Research Network: http://cancergenome.nih.gov/.

Although the knowledge concerning the dysregulation of the sialome and altered biosynthesis pathways is growing (16), the role of sialic acids in tumor development and immunity remains poorly understood. Yet, a better understanding of the impact of glycosylation changes in gynecological cancer has a high potential for the identification of diagnostic biomarkers and therapeutic targets. Glycan-based therapeutics may include specific sialyltransferase inhibitors (36), sialic acid mimetics (37), glycan-coated nanoparticles (19), glycan-modifying enzymes, as well as antibodies to glycans or their receptors (lectins) (12). The potential gain for patients with gynecologic cancers is high, but so are the challenges. The latter not only demand increased interdisciplinary efforts between clinicians and scientists, but also the improved training of glycoscientists (38) and the enhanced awareness of the biological implications of altered glycosylation on tumor biology and immunity.

## Author Contributions

SG and QH designed the study. QH, CS, and SG analyzed the data. Computational analysis of the data set was performed by QH. All authors had full access to the data, helped to draft the report or critically revised the draft, contributed to data interpretation, and reviewed and approved the final version of the report.

## Conflict of Interest Statement

The authors declare that the research was conducted in the absence of any commercial or financial relationships that could be construed as a potential conflict of interest.
